# Supplementary material for: Evidence for a Novel Gammaherpesvirus as the Putative Agent of Malignant Catarrhal Fever Disease in Roan Antelopes (Hippotragus equinus)
Source: Viruses. 2023 Feb 28;15(3):649. doi: 10.3390/v15030649 (PMC10051647; doi:10.3390/v15030649)
Supplement: Supplementary file 1 [file viruses-15-00649-s001.zip › viruses-2182071-supplementary.pdf]

Evidence for a novel gammaherpesvirus as the putative agent of malignant catarrhal fever disease in roan antelopes (*Hippotragus equinus*)

Technical appendix

Histopathology and immunohistochemistry

At necropsy, tissue samples were collected and fixed in 10% neutral buffered formalin for 24 hours. Afterwards, samples were trimmed, embedded in paraffin wax, cutted into 2 to 4 µm thick sections and mounted on SuperFrost®Plus slides (Glasbearbeitungswerke GmbH & Co. KG, Braunschweig, Germany). For light microscopy, hematoxylin and eosin (HE) staining was performed. In addition, immunohistochemistry for phenotypical characterization of inflammatory cells was performed on formalin-fixed and paraffin-embedded (FFPE) tissue of the rete mirabile. Therefore, following antibodies were used in their respective concentration (Table 1): an anti-CD3 antibody to detect T lymphocytes, an anti-CD20 antibody for the visualization of B lymphocytes, an anti-CD204 antibody as well as an anti-Iba1 antibody for the detection of macrophages. Table 1 provides an overview about used primary antibodies with their respective dilution and the pre-treatment. Immunohistochemistry was done as previously described [1]. Briefly, sections underwent deparaffinization and rehydration, followed by blocking of endogenous peroxidase and subsequent heat-induced antigen retrieval. Unspecific background staining was reduced by incubation with normal goat serum [diluted 1:5 in phosphate-buffered saline (PBS)] for one hour. Primary antibodies were applied in their respective concentration (Table 1) and incubated over night at 4°C. For negative controls, primary antibodies were substituted by normal rabbit serum (diluted 1:3000 in PBS) or Balb/c serum (diluted 1:1000 in PBS). Signal detection was achieved by applying biotinylated secondary antibodies directed against the respective species from which the primary antibody was generated, followed by signal amplification by the use of avidin-biotin-peroxidase complex (ABC, Vectastain ABC Kit Standard, Vector Laboratories, USA). Immunohistochemical reaction was detected using the chromogene 3,3-diaminobenzidine tetrahydrochloride (0.05%, Sigma Aldrich Chemie GmbH, Germany) with addition of 0.03% hydrogen peroxide. Subsequently, sections were counterstained with Mayer's hematoxylin (Roth C. GmbH & Co KG, Germany). Immunohistochemical investigation of the rete mirabile was analysed semiquantitatively. The graduation was determined as follows: minimal (0-3 immunopositive cells), mild (4 immunopositive cells to 33% immunopositive cells), moderate (33-66% immunopositive cells), marked (66-100% immunopositive cells).

Table S1. Used primary antibodies, clonality, species, dilution, antigen retrieval and company.

ANTIGEN	TARGET	CLONALITY, SPECIES	DILUTION	ANTIGEN RETRIEVAL	COMPANY
**CD3**	T lymphocyte	polyclonal, rabbit	1:200	citrate buffer, microwave	ThermoFisher Scientific
**CD20**	B lymphocyte	polyclonal, rabbit	1:400	citrate buffer, microwave	ThermoFisher Scientific
**CD204**	macrophage	monoclonal, mouse	1:250	citrate buffer, microwave	Biozol
**IBA1**	macrophage	polyclonal, rabbit	1:1000	citrate buffer, microwave	ThermoFisher Scientific

1. Störk, T.; de le Roi, M.; Haverkamp, A.-K.; Jesse, S.T.; Peters, M.; Fast, C.; Gregor, K.M.; Könenkamp, L.; Steffen, I.; Ludlow, M.; et al. Analysis of avian Usutu virus infections in Germany from 2011 to 2018 with focus on dsRNA detection to demonstrate viral infections. *Sci. Rep.* **2021**, *11*, 24191, doi:10.1038/s41598-021-03638-5.
